# Pulmonary tuberculosis associated acute fibrinous and organizing pneumonia: A case report and literature review

**DOI:** 10.1111/crj.13626

**Published:** 2023-05-08

**Authors:** Xiang Zhao, Yuan Cheng, Yan Xiong, Guangfa Wang

**Affiliations:** ^1^ Department of Respiratory and Critical Care Medicine Peking University First Hospital Beijing China; ^2^ Department of Pathology Peking University First Hospital Beijing China

**Keywords:** *Mycobacterium tuberculosis*, acute fibrinous and organizing pneumonitis, transbronchial cryobiopsy, tuberculosis

## Abstract

**Background:**

Acute fibrinous and organizing pneumonia (AFOP) is a rare histological interstitial pneumonia pattern characterized by patches of “fibrin balls” distributed within the alveoli and organizing pneumonia. Currently, there is no consensus on the diagnosis and treatment of this disease.

**Methods:**

We present the case of a 44‐year‐old male with AFOP secondary to Mycobacterium tuberculosis. We have further reviewed organizing pneumonia (OP) and AFOP caused by tuberculosis.

**Conclusion:**

Tuberculosis secondary to OP or AFOP is rare and challenging to diagnose. We need to constantly adjust the treatment plan based on the patient's symptoms, test results, and response to treatment in order to arrive at an accurate diagnosis and maximize treatment efficacy.

## Graphical abstract

Pulmonary tuberculosis presenting secondary OP and AFOP are rare. We present a case of 44‐year‐old male with AFOP secondary to Mycobacterium tuberculosis and searched existing literature relevant to OP and AFOP caused by tuberculosis.

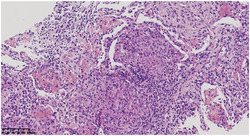

**Abbreviations**
AFOP: acute fibrinous and organizing pneumonitis
OP: organizing pneumonia
SOP: secondary organizing pneumonia
BAL: bronchoalveolar lavage
CT: computed tomography
DAD: Diffuse Alveolar Damage
MTB: *Mycobacterium tuberculosis*
NTM: non‐tuberculosis mycobacteria
PCR: polymerase chain reaction
LTBI: latent tuberculosis infection
IGRA: interferon‐gamma release assay
ATB: active tuberculosis

## 1 INTRODUCTION

Acute fibrinous and organizing pneumonia (AFOP), first described by Beasley in 2002, is a rare histological interstitial pneumonia pattern characterized by large amounts of fibrin deposition in the alveolar cavities and alterations associated with organizing pneumonia (OP).^1^ AFOP often termed a severe form of OP. A variety of unrelated miscellaneous infectious pathogens have been reported to cause secondary organizing pneumonia (SOP), including viruses, bacteria, fungi, or parasites.^2^ Rarely, it can also be caused by *Mycobacterium tuberculosis* (MTB). We describe a case of pulmonary tuberculosis associated AFOP. In addition, we reviewed the clinical characteristics of pulmonary tuberculosis related OP and AFOP to provide clinicians with experience in the diagnosis and treatment of this disease.^2^, ^3^, ^4^, ^5^, ^6^, ^7^

### 1.1 Case presentation

A 44‐year‐old male patient complained of cough with yellow mucous sputum for over 1 month and high fever for 2 weeks. His history included hepatitis B for 12 years, for which entecavir was administered regularly for 6 years until a negative report of hepatitis B virus DNA was obtained. Chest computed tomography (CT) showed bilateral patchy consolidation and ground‐glass opacity, after which he was admitted to the hospital. Sputum respiratory virus and fungal tests were negative. He was then treated with empirical antibiotics for approximately 2 weeks for suspected community‐acquired pneumonia. However, he did not recover and was transferred to our hospital for further treatment.

**FIGURE 1:**
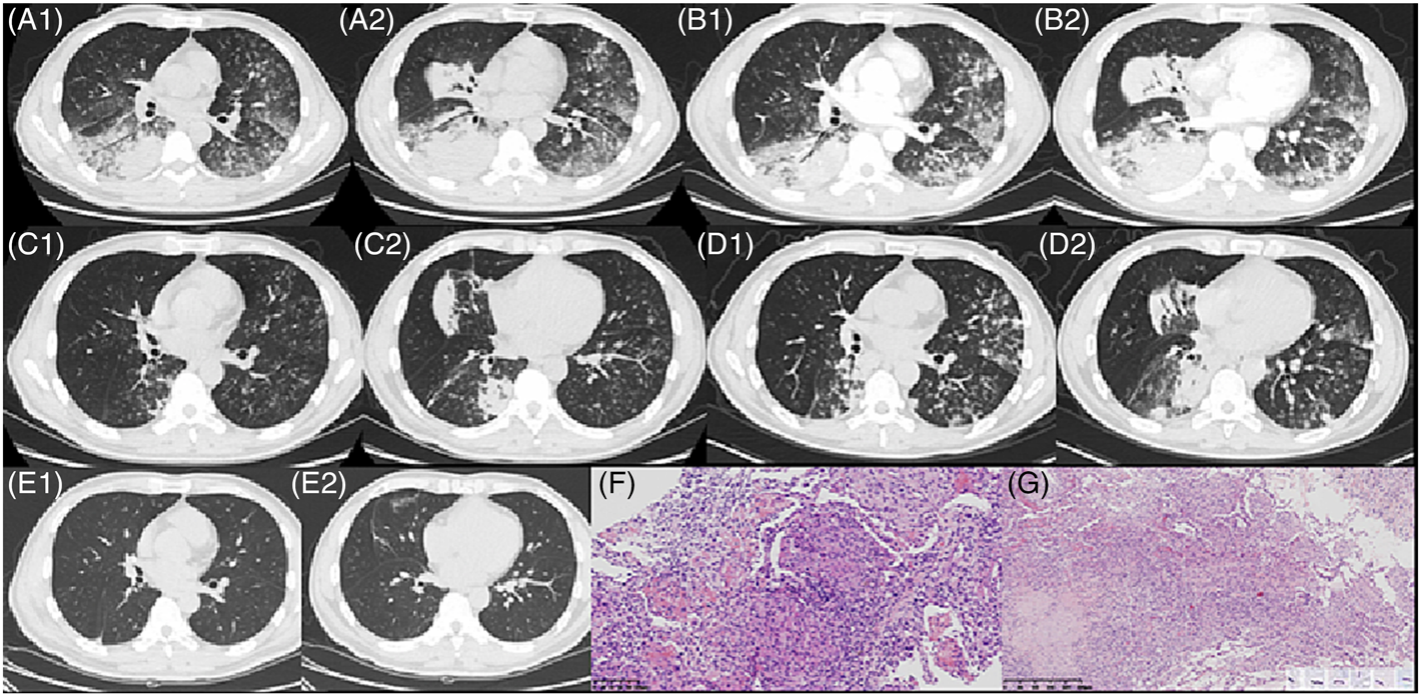
(A) Consolidation and ground glass opacity along the course of bronchioles, and multiple centrilobular nodules. (B) Progression of the pulmonary lesions. (C) The bilateral pulmonary lesions had absorbed, leaving only a few fibrous strands. (D) Multiple consolidations and ground glass nodules in the upper lobe of the left lung, the middle lobe of the right lung, and the lower lobe of both lungs progressed. (E) The lung consolidation, ground‐glass opacities, and multiple centrilobular nodules were absorbed. (F) Histopathology of percutaneous lung biopsy: acute fibrinous and organizing pneumonia: fibrin deposited in the alveolar spaces with proliferation of epithelioid histiocytes and development of granulation tissue. (G) Histopathology of transbronchial cryobiopsy: typical necrotizing granuloma with numerous acid‐fast bacilli detected in the necrotizing lesions (the small pictures inserted at the bottom right corner).

His blood pressure was 115/73 mmHg, heart rate was 99 beats/min, respiratory rate was 20 breaths/min, and temperature was 38.2°C. Arterial blood gas analysis revealed hypoxemia (partial pressure of oxygen [PaO_2_]/fraction of inspired oxygen [FiO_2_], 261.9 mmHg). A full blood count revealed the following parameters: Leukocyte count, 8.2 × 10^9^/L; neutrophils%, 80.2; hypersensitive C‐reactive protein, 102.27 mg/L; alanine aminotransferase, 61 U/L; aspartate aminotransferase, 109 U/L. Blood cultures, sputum cultures, and sputum acid‐fast bacilli were not detected. Nucleic acid detection of *Mycoplasma pneumoniae* or *Chlamydia pneumoniae*, MTB, influenza A and B, cytomegalovirus, Epstein–Barr virus, syncytial virus, adenovirus, and parainfluenza virus were all negative. Additional tests for mycoplasma antibody, Legionella antibody, cryptococcal antigen, G‐test, galactomannan antigen detection, antinuclear antibodies, extractable nuclear antigens, anti‐neutrophil cytoplasmic antibody, glomerular basement membrane antibody, and rheumatoid factor were within the normal limits. The CD4+ lymphocyte count was 375.66/μL, and Chlamydia IgM was negative, whereas Chlamydia IgG was120.1 RU/mL. T‐SPOT.TB test was positive, early secreted antigenic target‐6 (ESAT‐6) was 55SFCs, and culture filter protein‐10 (CFP‐10) was 8 SFCs. Bronchoalveolar lavage fluid (BALF) did not detect any bacteria, fungi, DNA viruses, or tuberculosis. The polymerase chain reaction (PCR) of MTB in BALF was also negative. The initial diagnosis was community‐acquired pneumonia, and the patient was treated with moxifloxacin and meropenem. However, the symptoms and CT scan findings (Figure 1A) improved minimally, and CT showed consolidation and ground‐glass opacities along the airway as well as multiple centrilobular nodules.

On day 3 of admission, oseltamivir was administered, moxifloxacin was replaced with levofloxacin, and ultrasound‐guided percutaneous lung biopsy (consolidation focus of the lower lobe of the right lung) was performed. On day 8, oseltamivir was discontinued after the completion of the treatment. On day 9, the symptoms did not improve after antibiotic treatment, and the pulmonary lesions progressed (Figure 1B). Histological examination showed that fibrin depositing in the alveolar spaces with proliferation of epithelioid histiocytes and development of granulation tissue. This morphology supported the diagnosis of acute fibrinous and organizing pneumonia (Figure 1F). On day 11, the high‐sensitivity C‐reactive protein level decreased, and the antibiotic was downgraded to piperacillin sulbactam; however, the patient still had intermittent fever. On day 14, the administration of methylprednisolone 80 mg was commenced. The patient's temperature returned to normal along with improvement in cough and expectoration. Antibiotics were stopped on day 15, and oxygen inhalation conditions gradually improved. Chest CT was performed on day 21 (Figure 1C), which showed that the bilateral pulmonary lesions had absorbed, leaving only a few fibrous strands. Steroids were tapered, and the patient was in a stable condition. On day 25, he continued to take 50 mg of oral prednisolone daily with outpatient follow‐up. The patient returned for fever on the twentieth day after discharge, and chest CT (Figure 1D) showed multiple consolidations and progression of ground‐glass density nodules in the upper lobe of the left lung, the middle lobe of the right lung, and the lower lobe of both lungs. The patient returned to the hospital and underwent transbronchial cryobiopsy using a rigid bronchoscope. Sputum acid‐fast bacilli were not detected. BALF still did not detect any bacteria, fungi, DNA viruses, or tuberculosis. The PCR of MTB in BALF was still negative. Lymphocytes accounted for 70% of the BALF. Transbronchial cryobiopsy pathology of the dorsal segment of the left lower lobe revealed AFOP and typical necrotizing granuloma with numerous acid‐fast bacilli (Ziehl–Neelsen staining) detected in the necrotizing lesions (Figure 1E). The patient was diagnosed with pulmonary tuberculosis, for which he was treated daily with 300 mg of isoniazid, 450 mg of rifampicin, 750 mg of ethambutol, and 1200 mg of pyrazinamide, and the glucocorticoid was quickly tapered off and discontinued within a month. Following the completion of the 6‐month anti‐tuberculosis drug regimen, his symptoms improved significantly. Three years later, a CT scan (Figure 1G) showed that the lung consolidation, ground‐glass shadow, and lobular core nodules were absorbed.

## 2 LITERATURE REVIEW

There are numerous causes of OP, including infection, inhalation, aspiration, diseases of the connective tissue, and extrapulmonary diseases. The investigation of the pathogenesis of SOP is a very difficult task.^1^ Based on our review of the literature, we found 12 cases reporting pulmonary tuberculosis presenting secondary OP and AFOP (Table 1).^2^, ^3^, ^4^, ^5^, ^6^, ^7^ Patient characteristics, clinical manifestations, laboratory examination, biopsy, steroid treatment, and prognosis were analyzed (Table 1). Taking the one case reported herein along with the other 12 reported worldwide, only two cases showed a positive result for the acid‐fast bacilli (AFB) smear of respiratory samples. MTB PCR of respiratory specimens was positive in seven out of nine cases (77.8%). AFB culture was positive in six out of nine cases (66.7%), three cases of *Mycobacterium* found in pathological tissues. Eleven cases lung tissue had OP histologically, and two cases lung tissue had AFOP histologically.^3^, ^4^, ^7^ While culture is the gold standard for diagnosing MTB, its positive rate is only 66.7%, which can be combined with various methods, including AFB smears, MTB PCR, MTB culture, and Ziehl–Neelsen staining in pathological tissues.

**TABLE 1:** Clinical characteristics, biopsy, steroid treatment, and prognosis of 13 cases of OP or AFOP associated with MTB.

Case	Year^Ref^	Age/sex	Symptom	Past medical history	Chest CT	BALF or sputum AFB smear	MTB PCR	AFB culture	Biopsy	Steroid treatment	Prognosis
1	2009^3^	F/27	cough, fever and right‐sided chest discomfort	AIDS	N/A	(−)	N/A	(+)	OP with AFB smear (+)	Not used	Dead because of fatal ARDS
2	2014^4^	M/80	fever, progressive dyspnea, and productive cough	without HIV infection	consolidation bilaterally	N/A	N/A	(−)	OP with AFB smear (+)	N/A	N/A
3	2015^5^	F/78	cough with sputum production	no previous significant medical history	multifocal consolidation in the right upper, right lower, and left lower lobes, with small nodules	(−)	(+)	N/A	OP	Not used	improvement
4	2015^5^	F/75	cough with yellowish sputum production and chest pain	no previous significant medical history	ground‐glass opacities and multifocal consolidation with air bronchogram in the posterior segment of the right upper lobe	(+)	(−)	N/A	OP	Not used	improvement
5	2016^6^	M/75	cough, mucus cough, moderate dyspnea, fever, joint pain and general discomfort	hypertension, atrial fibrillation, colonic polyp	bilateral lung infiltration and pleural effusion	N/A	N/A	(+)	OP	Methylprednisolone 40 mg/day	improvement
6	2020^2^	M/81	anorexia, fever, cough, and phlegm	no previous significant medical history	lobar consolidation in both upper lung fields, focal consolidation in right middle and right lower lobes, and bilateral pleural effusion	Sputum (−) BALF (+)	(+)	(−)	OP with small granuloma	Not used	improvement
7	2020^2^	M/72	cough, phlegm	no previous significant medical history	increased focal opacity in of left lower lung field on CXR	(−)	(+)	(+)	OP	received corticosteroid treatment	improvement
8	2020^2^	F/54	fever	no previous significant medical history	a mass measuring 3.2 × 2.8 cm in right upper lobe	(−)	(+)	(+)	OP	Not used	improvement
9	2020^2^	M/57	weakness and dyspnea	no previous significant medical history	multiple micronodules distributed randomly in both lungs, accompanied by cavitary nodules, irregular linear opacity, and patchy consolidation in both apices	(−)	(+)	N/A	OP	received corticosteroid treatment for a total of 6 months	improvement
10	2020^2^	M/78	fainting	no previous significant medical history	diffuse patchy consolidations and GGO with a crazy paving pattern in both lungs	(−)	(+)	(+)	OP	Not used	improvement
11	2020^2^	M/70	N/A	no previous significant medical history	solitary pulmonary nodule	(−)	(+)	(+)	OP	Not used	improvement
12	2014^7^	M64	Fever, dry cough and breathlessness	cerebral infarction, diabetes, and hypertension	bilateral massive lung consolidation lesion with scattered nodular opacities in the peripheral areas in the upper lobe of the left lung, and bilateral pleural effusion	(−)	(−)	(−)	AFOP, second pathological results: typical caseous necrosis and epithelioid cell granulomas with positive Ziehl–Neelsen staining result	80 mg twice daily intravenous drip, tapering schedule of methylprednisolone 12 mg twice daily	improvement

AFB acid fast bacillus, OP organizing pneumonia, AFOP acute fibrinous and organizing pneumonitis, N/A not available, ARDS acute respiratory distress syndrome.

In addition, distinguishing between MTB and non‐tuberculosis mycobacteria (NTM) species is a formidable obstacle. Traditional smear AFB staining produced positive results for both MTB and NTM. The T‐SPOT.TB may have cross‐reaction with *Mycobacterium kansasii*, *Mycobacterium marinum* and *Mycobacterium szulgai*, which may lead to false positives.^8^ The majority of NTM patients have obvious structural lung disease.^9^ Although these NTMs are also sensitive to rifampicin, they are all slow‐growing NTMs, which are very rare. It is unknown whether NTM can cause AFOP. A combination of our patients' symptoms, imaging manifestations, and T‐SPOT.TB test results, mycobacteria found on histopathology, and effective anti‐tuberculosis treatment, allowed us to diagnose secondary AFOP of mycobacteria, with a high probability of MTB infection. Because of the presence of heterogeneous lung lesions, it is possible that in the first biopsy there were already tuberculous lesions that were not sampled in the fine needle puncture. MTB have not been detected in the sputum or BALF because the patient received antibiotics, such as meropenem and moxifloxacin, prior to being diagnosed. The AFOP component of the lesion responded favorably to glucocorticoids, alleviating symptoms and consolidation images.

Of course, patients may also have latent tuberculosis infection (LTBI) with idiopathic AFOP, and MTB is activated following glucocorticoids. In a study of patients with rheumatic disease, 21.8% (349/1700) of patients were interferon‐gamma release assay (IGRA)‐positive. Eighteen (5.16%) IGRA‐positive patients and four (0.35%) IGRA‐negative patients had active tuberculosis after 2 years of follow‐up (*p* < 0.05).^10^ In the general population, IGRA can distinguish between active tuberculosis (ATB) and non‐ATB, and patients with ATB have higher T‐SPOT.TB results than patients with LTBI.^11^, ^12^ A T‐SPOT.TB test performed on our patient is strongly positive, and the diagnostic level reaches 90.28% when the threshold for T‐SPOT.TB positive ESAT‐6 and CFP‐10 is 40 sFU.^8^ We are more likely to diagnose pulmonary tuberculosis‐associated AFOP at the beginning of the disease. There is a significant correlation between T‐SPOT.TB results and the immune status of patients, and immunosuppression with LTBI leads to fewer positive IGRA results compared with the general population.^13^ IGRA is less sensitive in HIV‐positive individuals. The sensitivity of T.SPOT.TB in low‐ and middle‐income countries and high‐income countries is 72% and 94%, respectively, and the sensitivity of QFT‐GIT in low‐ and middle‐income countries and high‐income countries is 60% and 67%, respectively, according to a meta‐analysis.^14^ The patients with low CD4+ counts generally have decreases in sensitivity, especially those who are under 100/μL. The detection of LTBI among immunocompromised patients remains a challenge. At the moment, continuous IGRA is used to detect reactivated or newly acquired tuberculosis.^13^

The application of glucocorticoid in pulmonary tuberculosis is controversial. In our study, all 13 cases received anti‐tuberculosis treatment. In four cases, pulmonary lesions improved after administration of steroids together with anti‐tuberculosis treatment.^2^, ^6^, ^7^ Six cases improved after anti‐tuberculosis treatment without steroid treatment^2^, ^5^; an AIDS patient received antiretroviral therapy and anti‐tuberculosis treatment, eventually died of fatal ARDS.^3^ Comparing adjuvant glucocorticoid therapy with placebo for pulmonary tuberculosis has not yielded any high‐quality evidence of a reduction in mortality or sustained improvement in microbiological or clinical outcomes.^15^ The systematic evaluation published by Cochrane in 2014 included 18 trials, and because of the lack of large‐scale clinical trials using rifampicin in the treatment, there is no high‐quality evidence that glucocorticoid is beneficial for pulmonary tuberculosis patients in the context of antibacterial treatment of contemporary tuberculosis.^16^ A retrospective observational study assessed the effect of glucocorticoid on the 90‐day mortality rate of intensive care unit patients with pulmonary tuberculosis and acute respiratory failure, and the mortality rate decreased following analysis using treatment‐weighted methodologies.^17^ The majority of clinical practice guidelines recommend LTBI screening for patients who have initiated immunosuppression or are highly likely to initiate immunosuppression, as well as patients with immunosuppression owing to concurrent disease. Once active tuberculosis has been excluded, treatment for LTBI is advised.^18^ The American Rheumatology Society recommends that patients with rheumatoid arthritis complete at least 1 month of LTBI therapy before initiating or resuming treatment with TNF‐alpha inhibitors and other biological agents.^19^

In any case, OP is triggered by lung injury. Before being engulfed by alveolar macrophages, *M. tuberculosis* may first enter the alveolar epithelial cells. Macrophages gather around alveolar epithelial cells and destroy the alveolar epithelium, which is characterized by serous or serous cellulitis. The common result of injury is that a protein‐rich exudate leaks from the barrier into the alveolar space and is associated with migration of fibroblasts from the interstitium, differentiation of some fibroblasts into myofibroblasts, and formation of organizing fibroblastic tissue.^20^, ^21^ It is assumed that most inflammatory lung diseases have a period of intra‐alveolar fibrin development. In OP, leakage of coagulative proteins occurs during alveolar injury that will trigger an accumulation of fibrin from diminished fibrinolytic activity with fibroblast activation and proliferation ensuing giving the intra‐alveolar buds of granulation tissue (Masson bodies) appearance. However, in AFOP, fibrin is present in the form of fibrin “balls” with in the alveolar space. In fact, the components of fibrin globules are similar to those of transparent membranes formed during diffuse alveolar damage (DAD). The transparent membrane is attached to the alveolar surface, whereas the fibrin “balls” are contained in the alveolar spaces. After that, neutrophil levels decrease, and lymphocytes and macrophages are used as the main cellular components, eventually forming a granuloma.^2^, ^4^, ^5^, ^22^ In addition to typical tuberculous granuloma, it is speculated that fibroblasts are activated because of alveolar epithelial cell injury, thus showing the presence of organizing pneumonia.^2^

It is common for clinicians to assess secondary factors first, excluding predisposing factors such as infection, systemic diseases, or medication histories, as well as radiotherapy symptoms when treating OP. Pulmonary tuberculosis presenting secondary OP and AFOP are rare. Based on our case and previous literature, the positive rate of MTB detection by smear or culture of respiratory specimens is approximately 60%. Pathological diagnosis by fine needle aspiration or transbronchial lung biopsy may miss tumors, granulomas, and necrosis around OP. Therefore, we need to continuously adjust the treatment plan based on the patient's symptoms, test results, and response to treatment in order to obtain the correct diagnosis and optimal efficacy.

## AUTHOR CONTRIBUTIONS

Research ideas and study design, YC; Data acquisition, XZ; Data analysis/interpretation and statistical analysis, YC and XZ; Histological examination, YX; Supervision and mentorship, GFW. All authors read and approved the final manuscript.

## CONFLICT OF INTEREST STATEMENT

The authors declare that they have no competing interests.

## ETHICS APPROVAL AND CONSENT TO PARTICIPATE

Appropriate written informed consent was obtained from the patient for the publication of this case report and accompanying images. It was approved by the Clinical Research Ethics Committee of Peking University First Hospital and was implemented.

## Data Availability

All the relevant data and material are available from the corresponding author on reasonable request.
